# State of the Art on the Evidence Base in Cardiac Regenerative Therapy: Overview of 41 Systematic Reviews

**DOI:** 10.1155/2015/613782

**Published:** 2015-06-15

**Authors:** Mariangela Peruzzi, Elena De Falco, Antonio Abbate, Giuseppe Biondi-Zoccai, Isotta Chimenti, Marzia Lotrionte, Umberto Benedetto, Ronak Delewi, Antonino G. M. Marullo, Giacomo Frati

**Affiliations:** ^1^Department of Medico-Surgical Sciences and Biotechnologies, Sapienza University of Rome, Corso della Repubblica 79, 04100 Latina, Italy; ^2^VCU Pauley Heart Center, Virginia Commonwealth University, 821 West Franklin Street, Richmond, VA 23284, USA; ^3^Fondazione Eleonora Lorillard Spencer Cenci, 00185 Roma, Italy; ^4^Heart Failure and Cardiac Rehabilitation Unit, Columbus Integrated Complex, Via Giuseppe Moscati 31, 00168 Rome, Italy; ^5^Oxford Heart Center, Oxford University Hospital, Headley Way, Oxford OX3 9DU, UK; ^6^Academic Medical Center Amsterdam, Meibergdreef 9, 1105 AZ Amsterdam, Netherlands; ^7^Department of AngioCardioNeurology, IRCCS Neuromed, Via Atinense 18, 86077 Pozzilli, Italy

## Abstract

*Objectives*. To provide a comprehensive appraisal of the evidence from secondary research on cardiac regenerative therapy.* Study Design and Setting*. Overview of systematic reviews of controlled clinical trials concerning stem cell administration or mobilization in patients with cardiovascular disease.* Results*. After a systematic database search, we short-listed 41 reviews (660 patients). Twenty-two (54%) reviews focused on acute myocardial infarction (AMI), 19 (46%) on chronic ischemic heart disease (IHD) or heart failure (HF), 29 (71%) on bone marrow-derived stem-cells (BMSC), and 36 (88%) to randomized trials only. Substantial variability among reviews was found for validity (AMSTAR score: median 9 [minimum 3]; 1st quartile 9; 3rd quartile 10; maximum 11), effect estimates (change in ejection fraction from baseline to follow-up: 3.47% [0.02%; 2.90%; 4.22%; 6.11%]), and citations (Web of Science yearly citations: 4.1 [0; 2.2; 6.5; 68.9]). No significant association was found between these three features. However, reviews focusing on BMSC therapy had higher validity scores (*P* = 0.008) and showed more pronounced effect estimates (*P* = 0.002). Higher citations were associated with journal impact factor (*P* = 0.007), corresponding author from North America/Europe (*P* = 0.022), and inclusion of nonrandomized trials (*P* = 0.046).* Conclusions*. Substantial heterogeneity is apparent among these reviews in terms of quality and effect estimates.

## 1. Introduction

Heart failure (HF) represents a common final pathway for many patients with ischemic heart disease (IHD) or other chronic cardiac conditions [1], yet, the paradigm that the heart is a terminally differentiated organ without regenerative capacity has been challenged several times [2, 3]. In addition, we may also envision means to implant by various routes autologous or heterologous stem cells which may eventually regenerate the heart either directly, that is, by differentiating into cardiomyocytes, or indirectly, that is, by exerting local humoral effects which activate endogenous cardiac regenerative potential through paracrine enhancement, angiogenic, or anti-inflammatory actions [4–8]. Accordingly, several clinical trials, mostly phases 2 and 3, have been conducted on cardiac regenerative therapy [9, 10]. However, uncertainty persists given the limitations of existing studies and the difficulty in standardizing such regenerative therapy protocols [11].

Systematic reviews are commonly used for evidence synthesis, pooling original studies (i.e., primary research), in order to increase precision, test for homogeneity and consistency, and explore the impact of moderators [12]. Accordingly, systematic reviews are often defined as secondary research. Another layer of evidence synthesis has been recently advocated, based on the search, analysis, and interpretation of systematic reviews. This additional level of evidence synthesis is called tertiary research, and typical tools consist of umbrella reviews (i.e., overviews of reviews) and metaepidemiologic studies [13]. Umbrella reviews consist indeed of explicit and systematic overviews of previously published systematic reviews and meta-analyses focusing at large on a clinical topic. They represent a relatively novel and remarkably efficient tool to synthesize the evidence base on a specific clinical topic and highlight key discrepancies. Prior efforts at reconciling discrepancies between meta-analyses on the same topic have occurred [14], but to date there is no comprehensive overview of reviews on cardiac regenerative therapy. Accordingly, we aimed to undertake such a scholarly endeavor, with three specific goals: (a) appraising review quality and its predictors; (b) appraising consistency in effect estimates and their predictors; and (c) quantifying scholarly citations and their predictors.

## 2. Materials and Methods

### 2.1. Design

This review was performed and is reported in keeping with the Preferred Items for Systematic Reviews and Meta-Analyses (PRISMA) recommendations, but without a specific a priori protocol [15]. All reviewing activities were performed by two independent and experienced reviewers, with divergences resolved after consensus.

### 2.2. Search and Selection

The search for systematic reviews and meta-analyses on cardiac regenerative medicine was based on a dedicated search of The Cochrane Library and on an explicit query in MEDLINE/PubMed, last updated on July 26, 2014, performed with the Clinical Queries tool using the following as string: (cardiac OR coronary OR heart OR myocardi^∗^) AND ((growth AND factor^∗^) OR (regeneration OR regenerative OR stem OR cell^∗^) AND therapy).

After screening at the title and abstract level, the full text of potentially eligible reviews was analyzed leading to the final inclusion only of systematic reviews of clinical trials including statistical pooling of quantitative estimates (i.e., meta-analysis) of prognostic, cardiac function, or symptomatic data stemming from clinical trials on cardiac regenerative therapy, including stem cell administration or mobilization with growth factors, stimulating factors, or gene therapy. Specifically, fatal and nonfatal clinical events (including rehospitalizations) were considered as prognostic endpoints, parameters of left ventricular function, dimension, or viability, as well as cardiopulmonary function parameters, were considered as cardiac remodeling and/or cardiac function parameters, and functional class or prevalence of specific symptoms were considered as symptomatic endpoints. Reference lists of included studies were also screened for additional suitable articles. In addition, we excluded studies published in non-English language.

### 2.3. Abstraction, Quality Appraisal, Effect Estimate, and Citation Count

We extracted a comprehensive set of key study, patient, procedural, outcome, and validity features from the included studies. We explicitly appraised review quality with A Measurement Tool to Assess Systematic Reviews (AMSTAR) tool, which is an externally validated tool for the evaluation of the internal validity of a systematic review [16]. Effect estimates from included reviews were extracted as point estimates and 95% confidence intervals, with standard errors back-computed according to normal approximation. Finally, we obtained citation counts and computed yearly citations for each shortlisted review published before November 15, 2013, from Web of Science, Scopus, and Google Scholar. Specifically, yearly citations were computed as total citations divided by the time elapsed between publication and citational database analysis. Citation counts were last updated on November 15, 2014.

### 2.4. Analysis

Continuous variables are reported as median (minimum; 1st quartile; 3rd quartile; maximum). Categorical variables are reported as count (percentage). Bivariate analyses were based on Student's *t*-test, ANOVA, linear regression, or Fisher exact test. Multifactorial regression (i.e., regression with multiple independent variables but a single dependent variable) was based on linear or logistic regression with backward stepwise selection (*P* for removal 0.10). As an additional analysis, we explored the association between moderators and outcomes of interest with random effects metaregression, using as weights the within-review standard errors. Heterogeneity in effect estimates was also explored with Cochran *Q* test and *I* ^2^. To take into account the skewness in yearly citation counts, these were used for regression analysis after natural logarithm transformation. Statistical significance was set at the two-tailed 0.05 level, with *P* values unadjusted for multiplicity reported throughout. Computations were performed with Stata 13 (StataCorp, College Station, TX, USA).

## 3. Results and Discussion

**Figure 1:**
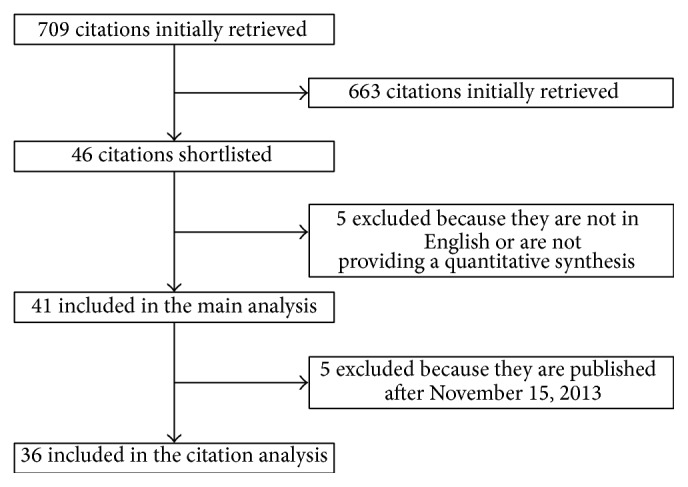
Review profile.

**Figure 2:**
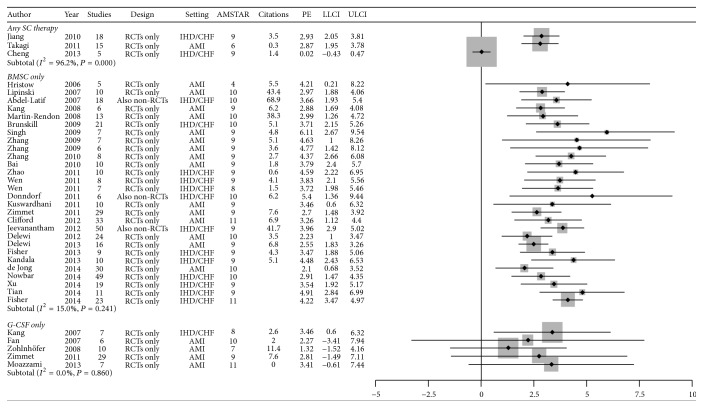
Forest plot for improvement in cardiac function (change in left ventricular ejection fraction). AMI = acute myocardial infarction; AMSTAR = A Measurement Tool to Assess Systematic Reviews; BMSC = bone marrow-derived stem cell; CHD = congestive heart failure; G-CSF = granulocyte-colony stimulating factor; IHD = ischemic heart disease; LLCI = lower limit of the 95% confidence interval; PE = point estimate; RCT = randomized clinical trial; SC = stem cell; ULCI = upper limit of the 95% confidence interval.

**Figure 3:**
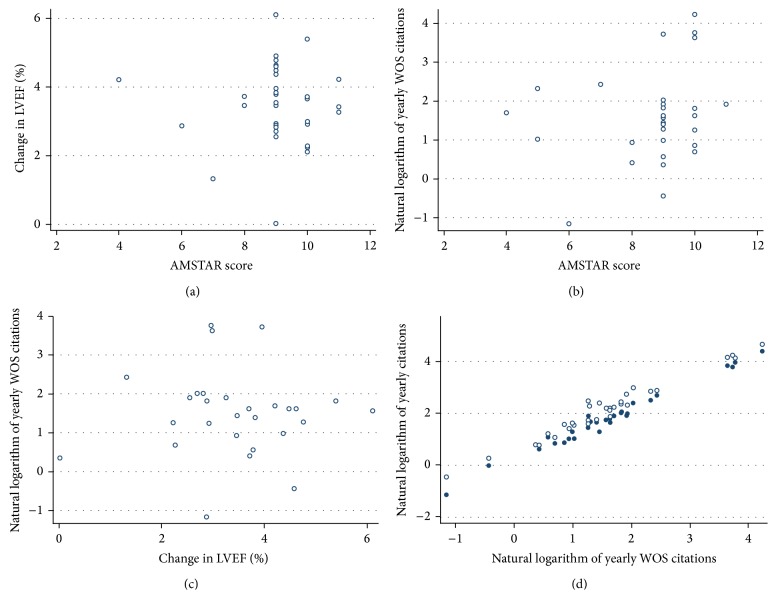
Association between review quality (measured with A Measurement Tool to Assess Systematic Reviews (AMSTAR) score), effect estimates for improvement in cardiac function (change in left ventricular ejection fraction (LVEF)), and yearly scholarly citations: (a) AMSTAR score and change in LVEF; (b) AMSTAR score and yearly citations in Web of Science (WOS); (c) change in LVEF and yearly citations in WOS; (d) yearly citations in WOS and yearly citations in Scopus (dots) or Google Scholar (circles).

From a total of 709 citations, 41 were finally included in the main analysis for validity and effect estimates and 36 in the citation analysis (online references; Table 1; see Table 1S, Table 2S, and Table 3S in Supplementary Material available online at http://dx.doi.org/10.1155/2015/613782; Figure 1). These were published between 2006 and 2014 and included a median of 10 studies (minimum 2; 1st quartile 7; 3rd quartile 18; maximum 50) and 660 patients (179; 412; 985; 2,625) (Table 1). Most (36 [88%]) reviews included only randomized clinical trials (RCT) but 6 (12%) did include both RCTs and non-RCTs, typically using for the latter type of study unadjusted effect estimates, while 22 (54%) focused on acute myocardial infarction (AMI), 19 (46%) focused on chronic ischemic heart disease (IHD) or heart failure (HF), and 29 (71%) limited their scope to bone marrow-derived stem cells (BMSC). Review validity was typically high (average of 9 out of 11) but remarkably variable (3; 9; 10; 11). Out of all the reviews, two (5%) reviews suggested a beneficial effect on symptoms, prognostic benefits were reported by 12 (29%) reviews, and 32 (78%) reviews reported on cardiac function parameters or signs of cardiac disease. Quantitative effect estimates were also variable (median change in LVEF = 3.47% [0.02%; 2.90%; 4.22%; 6.11%]; *P* for heterogeneity ranging from <0.001 to 0.880, *I* ^2^ ranging from 96% to 0%), having no significant association with review validity or year of publication (Figures 2 and 3), despite an improvement in precision over the years (linear regression of the standard error of the change in LVEF versus year: coefficient = −0.10, standard error = 0.04, *P* = 0.019). Yearly scholarly citations were on average 4.1 (0; 2.2; 6.5; 68.9) in Web of Science, 5.1 (0; 2.5; 7.4; 81.8) in Scopus, and 7.3 (0; 3.2; 11.3; 105.2) in Google Scholar and were also not apparently associated with review quality or quantitative effect estimates.

A more formal analysis of moderators or features potentially explaining the wide variability in AMSTAR scores, change in LVEF, and yearly citations was conducted, appraising at bivariate analysis an exhausting list of variables: year of publication, journal impact factor, journal subject, number of authors, country of corresponding author, specialty of corresponding author, review design, studies included, patients included, type of included studies, clinical setting, type of therapy, impact on prognosis, cardiac function parameters/signs or symptoms, funding, conflict of interests, random effects for pooling, subgroup analyses, and metaregression (Table 2). Despite this comprehensive set of analyses, only focus on BMSC therapy was found to be significantly associated with AMSTAR scores (*P* = 0.021) and change in LVEF (*P* = 0.002). Conversely, scholarly citations were significantly and positively predicted by journal impact factor (0.007) and corresponding author from North America/Europe (0.022). Finally, and rather surprisingly, reviews including RCTs as well as nonrandomized trials received more yearly citations than meta-analyses limited to RTCs only (*P* = 0.046). Bivariate and multivariable regression analyses were confirmed at random effects meta-regression, with very similar results in terms of statistical magnitude and direction.

The field of cardiac regenerative therapy has seen significant changes since the paradigm that the heart is a terminally differentiated organ was challenged. The amount of human and economic resources invested to date in this field has been extremely large, yet conclusive trials in cardiac regenerative therapy have not been completed, and the therapy has not affected clinical practice in any measurable way [1, 3, 17, 18].

There are several explanations for these setbacks, including our limited understanding of physiology and pathophysiology of cardiomyocytes, as well as stem cells, difficulties in standardizing protocols for cell harvesting and expansion, complexity in culture standardization, issues in identifying the correct patient subsets who are most likely to benefit from cardiac regenerative therapy, and difficulties in conducting high quality clinical trials [19–22]. Indeed, most studies to date have been preclinical ones, either focused on activity and efficacy (phase 2) or focused on initial evidence of effectiveness (phase 3), with single-center design and obvious limitations in internal and external validity.

Despite this, or possibly exactly for this reason, cardiac regenerative therapy has been the focus of intense research synthesis efforts, with the publication of many systematic reviews and meta-analyses including basic research as well as human clinical studies. Nonetheless, the heterogeneity between primary studies has been mirrored by heterogeneous results and conclusions among available meta-analyses, further complicating decision making on this topic. Overviews of reviews and umbrella reviews, together with metaepidemiologic studies, are now commonly used to summarize and appraise the evidence base on specific clinical topics, while maintaining the ability to focus on the single piece of evidence being lumped together with similar ones [12, 23].

Our present umbrella review provides indeed a concise yet comprehensive appraisal of 41 systematic reviews of clinical trials on cardiac regenerative therapy, pooling together dozens of studies and thousands of patients (not discounting duplicates). We found indeed that the evidence base on G-CSF and Ad5FGF-4 suggests no net effect on clinical events, cardiac remodeling, or symptoms in patients with AMI, chronic IHD, or HF. Most reviews that have focused on BMSC administration, instead, suggest some net benefit on cardiac remodeling, even if this is not large in absolute terms and might thus be not clinically relevant. In addition, some studies reported also a favorable effect on prognosis and symptoms, but these estimates may be undermined by reporting bias or other confounding factors. We have also highlighted the huge variability in review quality, effect estimates, and scholarly citations. In addition, we have explored potential moderators of these features, emphasizing the fact that type of therapy, journal impact factor, geographic area of corresponding authors from North America or Europe, and focus on non-RCTS are the only meaningful predictors for one or more of such features.

Indeed, a looming presence in this scholarly field is the concept of discrepancies, biases, and unreliable data, which may apply to some or many of the potentially eligible primary studies. For instance, it is troubling that de Jong et al. have not shown any benefit of BMSC on MRI-derived cardiac function parameters at odds with other studies using mainly echocardiographic data, which are considered much more prone to observer bias. Interpretation and decision making become even more complicated in light of the discrepancies in autologous bone marrow stem cell trials and enhancement of ejection fraction (DAMASCENE) analysis by Nowbar and colleagues, which disclosed over 600 discrepancies among 133 study reports stemming from 49 clinical trials on BMSC, concluding that the more the discrepancies, the greater the apparent effect of BMSC on LVEF [24]. All these lessons learned since the earliest clinical trials, mostly based on extracardiac cell sources, should hopefully pave the way for more accurate and critical design and interpretation of the latest trials for cardiac regenerative medicine, exploiting cardiac resident populations of progenitor cells.

These findings suggest that researchers, clinicians, funders, and patients should exert great caution in interpreting results of studies on cardiac regenerative therapy, to avoid an overoptimistic interpretation, as well as undue funding of less than promising research avenues. In addition, our results call for greater attention to details and internal validity and for a more cautious stance to any report of overwhelming clinical, remodeling, or symptomatic benefit of cardiac regenerative therapy [25]. The role of funding agencies (either private or public) will continue to remain crucial, with the need for careful assessment of the true merits of future preclinical and clinical research protocols, in order to minimize the risk of biased findings and hopefully bridge the gap between potential and actual benefits for patients. Having also early phase studies designed as rigorous placebo- or sham-controlled RCTs might be beneficial, in order to minimize the risk of bias. However, providing more formal recommendations is beyond the scope of the umbrella review design of the present work.

Despite our extensive effort, this overview of reviews has many limitations, and it should be interpreted with caution, as any form of nonprimary research. In particular, we limited our search to MEDLINE/PubMed and articles in English, and our work carries the risk, as any similar effort, of multiplicity and ecological fallacy [26, 27]. Conversely, the choice of using the Fisher exact test for bivariate analysis of categorical variables may have led to reduced statistical precision and power. However, we chose this analytical strategy in order to minimize the risk of type I error and multiplicity. Finally, while the reader might be tempted by the idea of performing a meta-analysis of the included meta-analyses, such analysis would be fraught by a major tautology issue, with some studies providing effect estimates larger than the real ones because they had been included in several reviews. Indeed, despite differences in the search and ultimate selection of studies, the core set of included trials was common to most meta-analyses (Figure 1S). Accordingly, no such analysis was performed.

## 4. Conclusions

Substantial heterogeneity is apparent among reviews on cardiac regenerative therapy completed to date in terms of quality and effect estimates, with no improvement in such key dimensions over time and a disturbing lack of or negative association between review quality and subsequent scholarly impact.

## Supplementary Material

In the supplementary material we sorted the reviews included in the study by different features and in the specific:
Table 1S: Reviews included in the study sorted by Journal, Journal Impact Factor, Year of Publication, PMID, Number of studies included and patients included.
Table 2S: Reviews included in the study sorted by year of publication, clinical setting, therapy accomplished, country of corresponding author and number of authors.
Table 3S: Reviews included in the study sorted by First author (acronym), Year of publication, AMSTAR score, clinical findings, Effect estimates for LVEF, Effect estimates for death and Yearly WOS citations.
Figure 1S: Representative trend of the included studies over time.

## Figures and Tables

**Table 1:** Key features of the 41 included reviews.

Feature	Median or count (%)	Minimum	1st quartile	3rd quartile	Maximum
Year of publication	2011	2006	2009	2013	2014
Authors	6	2	4	8	26
North American/European corresponding author	21	—	—	—	—
Studies included	10	2	7	18	50
Patients included	660	179	412	985	2,625
Randomized trials only	36	—	—	—	—
Type of setting					
Acute myocardial infarction	22 (54%)	—	—	—	—
Chronic ischemic heart disease or heart failure	19 (46%)	—	—	—	—
Type of therapy					
Any stem cell	6 (15%)	—	—	—	—
Bone marrow-derived stem cell	29 (71%)	—	—	—	—
Granulocyte-colony stimulating factor	5 (12%)	—	—	—	—
Other	1 (2%)	—	—	—	—
Patient-level meta-analysis	2 (5%)	—	—	—	—
Random effects	36 (88%)				
Small study effect testing	34 (83%)				
Subgroup analysis	38 (93%)	—	—	—	—
Metaregression	8 (20%)	—	—	—	—
Conflict of interests	2 (5%)	—	—	—	—
Funding	27 (66%)	—	—	—	—
A Measurement Tool to Assess Systematic Reviews (AMSTAR) score	9	3	9	10	11
Favorable findings on prognosis	12 (29%)	—	—	—	—
Favorable findings on symptoms	2 (5%)	—	—	—	—
Favorable findings on cardiac function parameters or signs	32 (78%)	—	—	—	—
Change in left ventricular ejection fraction	3.47%	0.02%	2.90%	4.22%	6.11%
Yearly Web of Science citations	4.1	0	2.2	6.5	68.9
Yearly Scopus citations	5.1	0	2.5	7.4	81.8
Yearly Google Scholar citations	7.3	0	3.2	11.3	105.2

**Table 2:** Bivariate and multivariable analysis for review quality, effect estimates, and scholarly citations.

Dependent variable	Independent variable(s)	Bivariate analysis^*^			Multivariable analysis^†^		
		Coefficient	Standard error	*P*	Coefficient	Standard error	*P*
AMSTAR score	Year of publication	0.27	0.11	0.022	0.21	0.11	0.052
	BMSC therapy	1.58	0.57	0.009	1.36	0.56	0.021

Change in LVEF (%)	BMSC therapy	1.41	0.42	0.002	—	—	—

Yearly Web of Science citations	Journal impact factor	0.85	0.20	<0.001	0.56	0.20	0.007
	Authors	0.10	0.04	0.034	—	—	—
	Corresponding author from North America or Europe	1.32	0.33	<0.001	0.77	0.32	0.022
	Studies included	0.04	0.02	0.037	—	—	—
	Patients included (×100 people)	0.07	0.03	0.030	—	—	—
	RCTs only	−1.35	0.56	0.023	−0.90	0.43	0.046

^∗^Only independent variables significantly (*P* < 0.05) associated with the dependent variable of interest are reported, but all the following variables were tested: year of publication, authors, North American/European corresponding author, studies included, patients included, RCTs only, type of setting, BMSC therapy, patient-level design, random effects analysis, small study effect testing, subgroup analysis, metaregression, conflict of interests, and funding; ^†^a multivariable linear regression model with backward stepwise selection (*P* for exit 0.10) was used, including in the model all variables significantly (*P* < 0.05) associated with the dependent variable at bivariate analysis; A Measurement Tool to Assess Systematic Reviews (AMSTAR) score; BMSC = bone marrow-derived stem cell; LVEF = left ventricular ejection fraction; RCT = randomized clinical trial.
