# Supplementary material for: State of the Art on the Evidence Base in Cardiac Regenerative Therapy: Overview of 41 Systematic Reviews
Source: Biomed Res Int. 2015 Jun 15;2015:613782. doi: 10.1155/2015/613782 (PMC4484838; doi:10.1155/2015/613782)
Supplement: Supplementary file 1 — In the supplementary material we sorted the reviews included in the study by different features and in the specific: Table 1S: Reviews included in the study sorted by Journal, Journal Impact Factor, Year of Publication, PMID, Number of studies included and patients included. Table 2S: Reviews included in the study sorted by year of publication, clinical setting, therapy accomplished, country of corresponding author and number of authors. Table 3S: Reviews included in the study sorted by First author (acronym), Year of publication, AMSTAR score, clinical findings, Effect estimates for LVEF, Effect estimates for death and Yearly WOS citations. Figure 1S: Representative trend of the included studies over time. [file 613782.f1.pdf]

Supplementary appendix to Biondi-Zoccai et al, State of the art on the evidence base in cardiac regenerative therapy: overview of 41 systematic reviews

ONLINE REFERENCES

Abdel-Latif A, Bolli R, Tleyjeh IM, Montori VM, Perin EC, Hornung CA, Zuba-Surma EK, Al-Mallah M, Dawn B. Adult bone marrow-derived cells for cardiac repair: a systematic review and meta-analysis. Arch Intern Med 2007;167:989-97.

Alvarez PA, Schwarz ER, Ramineni R, Myatt P, Barbin C, Boissonnet C, Phan A, Maggioni A, Barbagelata A. Periprocedural adverse events in cell therapy trials in myocardial infarction and cardiomyopathy: a systematic review. Clin Res Cardiol 2013;102(1):1-10.

Bai Y, Sun T, Ye P. Age, gender and diabetic status are associated with effects of bone marrow cell therapy on recovery of left ventricular function after acute myocardial infarction: a systematic review and meta-analysis. Ageing Res Rev 2010;9:418-23.

Brunskill SJ, Hyde CJ, Doree CJ, Watt SM, Martin-Rendon E. Route of delivery and baseline left ventricular ejection fraction, key factors of bone-marrow-derived cell therapy for ischaemic heart disease. Eur J Heart Fail 2009;11:887-96.

Cheng K, Wu F, Cao F. Intramyocardial autologous cell engraftment in patients with ischaemic heart failure: a meta-analysis of randomised controlled trials. Heart Lung Circ 2013;22:887-94.

Clifford DM, Fisher SA, Brunskill SJ, Doree C, Mathur A, Clarke MJ, Watt SM, Martin-Rendon E. Long-term effects of autologous bone marrow stem cell treatment in acute myocardial infarction: factors that may influence outcomes. PLoS One 2012;7:e37373.

de Jong R, Houtgraaf JH, Samiei S, Boersma E, Duckers HJ. Intracoronary stem cell infusion after acute myocardial infarction: a meta-analysis and update on clinical trials. Circ Cardiovasc Interv 2014;7:156-67.

Delewi R, Hirsch A, Tijssen JG, Schächinger V, Wojakowski W, Roncalli J, Aakhus S, Erbs S, Assmus B, Tendera M, Goekmen Turan R, Corti R, Henry T, Lemarchand P, Lunde K, Cao F, Huikuri HV, Sürder D, Simari RD, Janssens S, Wollert KC, Plewka M, Grajek S, Traverse JH, Zijlstra F, Piek JJ. Impact of intracoronary bone marrow cell therapy on left ventricular function in the setting of ST-segment elevation myocardial infarction: a collaborative meta-analysis. Eur Heart J 2014;35:989-98.

Delewi R, Andriessen A, Tijssen JG, Zijlstra F, Piek JJ, Hirsch A. Impact of intracoronary cell therapy on left ventricular function in the setting of acute myocardial infarction: a meta-analysis of randomised controlled clinical trials. Heart 2013;99:225-32.

Donndorf P, Kundt G, Kaminski A, Yerebakan C, Liebold A, Steinhoff G, Glass A. Intramyocardial bone marrow stem cell transplantation during coronary artery bypass surgery: a meta-analysis. J Thorac Cardiovasc Surg 2011;142:911-20.

Fan L, Chen L, Chen X, Fu F. A meta-analysis of stem cell mobilization by granulocyte colony-stimulating factor in the treatment of acute myocardial infarction. Cardiovasc Drugs Ther 2008;22:45-54.

Fisher SA, Dorée C, Brunskill SJ, Mathur A, Martin-Rendon E. Bone Marrow Stem Cell Treatment for Ischemic Heart Disease in Patients with No Option of Revascularization: A Systematic Review and Meta-Analysis. PLoS One 2013;8:e64669.

Fisher SA, Brunskill SJ, Doree C, Mathur A, Taggart DP, Martin-Rendon E. Stem cell therapy for chronic ischaemic heart disease and congestive heart failure. Cochrane Database Syst Rev 2014;4:CD007888.

Henry TD, Grines CL, Watkins MW, Dib N, Barbeau G, Moreadith R, Andrasfay T, Engler RL. Effects of Ad5FGF-4 in patients with angina: an analysis of pooled data from the AGENT-3 and AGENT-4 trials. J Am Coll Cardiol 2007;50:1038-46.

Hristov M, Heussen N, Schober A, Weber C. Intracoronary infusion of autologous bone marrow cells and left ventricular function after acute myocardial infarction: a meta-analysis. J Cell Mol Med 2006;10:727-33.

Ince H, Valgimigli M, Petzsch M, de Lezo JS, Kuethe F, Dunkelmann S, Biondi-Zoccai G, Nienaber CA. Cardiovascular events and re-stenosis following administration of G-CSF in acute myocardial infarction: systematic review and meta-analysis. Heart 2008;94:610-6.

Jeevanantham V, Butler M, Saad A, Abdel-Latif A, Zuba-Surma EK, Dawn B. Adult bone marrow cell therapy improves survival and induces long-term improvement in cardiac parameters: a systematic review and meta-analysis. Circulation 2012;126:551-68.

Jiang M, He B, Zhang Q, Ge H, Zang MH, Han ZH, Liu JP, Li JH, Zhang Q, Li HB, Jin Y, He Q, Gong XR, Yin XY. Randomized controlled trials on the therapeutic effects of adult progenitor cells for myocardial infarction: meta-analysis. Expert Opin Biol Ther 2010;10:667-80.

Jiang M, Mao J, He B. The effect of bone marrow-derived cells on diastolic function and exercise capacity in patients after acute myocardial infarction. Stem Cell Res 2012;9:49-57.

Kandala J, Upadhyay GA, Pokushalov E, Wu S, Drachman DE, Singh JP. Meta-analysis of stem cell therapy in chronic ischemic cardiomyopathy. Am J Cardiol 2013;112:217-25.

Kang S, Yang Y, Li CJ, Gao R. Effectiveness and tolerability of administration of granulocyte colony-stimulating factor on left ventricular function in patients with myocardial infarction: a meta-analysis of randomized controlled trials. Clin Ther 2007;29:2406-18.

Kang S, Yang YJ, Li CJ, Gao RL. Effects of intracoronary autologous bone marrow cells on left ventricular function in acute myocardial infarction: a systematic review and meta-analysis for randomized controlled trials. Coron Artery Dis 2008;19:327-35.

Kuswardhani RA, Soejitno A. Bone marrow-derived stem cells as an adjunctive treatment for acute myocardial infarction: a systematic review and meta-analysis. Acta Med Indones 2011;43:168-77.

Li N, Yang YJ, Zhang Q, Jin C, Wang H, Qian HY. Stem cell therapy is a promising tool for refractory angina: a meta-analysis of randomized controlled trials. Can J Cardiol 2013;29:908-14.

Lipinski MJ, Biondi-Zoccai GG, Abbate A, Khianey R, Sheiban I, Bartunek J, Vanderheyden M, Kim HS, Kang HJ, Strauer BE, Vetrovec GW. Impact of intracoronary cell therapy on left ventricular function in the setting of acute myocardial infarction: a collaborative systematic review and meta-analysis of controlled clinical trials. J Am Coll Cardiol 2007;50:1761-7.

Martin-Rendon E, Brunskill SJ, Hyde CJ, Stanworth SJ, Mathur A, Watt SM. Autologous bone marrow stem cells to treat acute myocardial infarction: a systematic review. Eur Heart J 2008;29:1807-18.

Moazzami K, Roohi A, Moazzami B. Granulocyte colony stimulating factor therapy for acute myocardial infarction. Cochrane Database Syst Rev 2013;5:CD008844.

Nowbar AN, Mielewczik M, Karavassilis M, Dehbi HM, Shun-Shin MJ, Jones S, Howard JP, Cole GD, Francis DP; DAMASCENE writing group. Discrepancies in autologous bone marrow stem cell trials and enhancement of ejection fraction (DAMASCENE): weighted regression and meta-analysis. BMJ. 2014;348:g2688.

Singh S, Arora R, Handa K, Khraisat A, Nagajothi N, Molnar J, Khosla S. Stem cells improve left ventricular function in acute myocardial infarction. Clin Cardiol 2009;32:176-80.

Sun L, Zhang T, Lan X, Du G. Effects of stem cell therapy on left ventricular remodeling after acute myocardial infarction: a meta-analysis. Clin Cardiol 2010;33:296-302.

Takagi H, Umemoto T. Intracoronary stem cell injection improves left ventricular remodeling after acute myocardial infarction: an updated meta-analysis of randomized trials. Int J Cardiol 2011;151:226-8.

Tian T, Chen B, Xiao Y, Yang K, Zhou X. Intramyocardial autologous bone marrow cell transplantation for ischemic heart disease: a systematic review and meta-analysis of randomized controlled trials. Atherosclerosis 2014;233:485-92.

Wen Y, Meng L, Ding Y, Ouyang J. Autologous transplantation of blood-derived stem/progenitor cells for ischaemic heart disease. Int J Clin Pract 2011;65:858-65.

Wen Y, Meng L, Xie J, Ouyang J. Direct autologous bone marrow-derived stem cell transplantation for ischemic heart disease: a meta-analysis. Expert Opin Biol Ther 2011;11:559-67.

Xu R, Ding S, Zhao Y, Pu J, He B. Autologous Transplantation of Bone Marrow/Blood-Derived Cells for Chronic Ischemic Heart Disease: A Systematic Review and Meta-analysis. Can J Cardiol 2014 Jan 23. [Epub ahead of print].

Zhang S, Sun A, Xu D, Yao K, Huang Z, Jin H, Wang K, Zou Y, Ge J. Impact of timing on efficacy and safetyof intracoronary autologous bone marrow stem cells transplantation in acute myocardial

infarction: a pooled subgroup analysis of randomized controlled trials. Clin Cardiol 2009;32:458-66.

Zhang SN, Sun AJ, Ge JB, Yao K, Huang ZY, Wang KQ, Zou YZ. Intracoronary autologous bone marrow stem cells transfer for patients with acute myocardial infarction: a meta-analysis of randomised controlled trials. Int J Cardiol 2009;136:178-85.

Zhang C, Sun A, Zhang S, Yao K, Wu C, Fu M, Wang K, Zou Y, Ge J. Efficacy and safety of intracoronary autologous bone marrow-derived cell transplantation in patients with acute myocardial infarction: insights from randomized controlled trials with 12 or more months follow-up. Clin Cardiol 2010;33:353-60.

Zhao Q, Ye X. Additive value of adult bone-marrow-derived cell transplantation to conventional revascularization in chronic ischemic heart disease: a systemic review and meta-analysis. Expert Opin Biol Ther 2011;11:1569-79.

Zimmet H, Porapakkham P, Porapakkham P, Sata Y, Haas SJ, Itescu S, Forbes A, Krum H. Short- and long-term outcomes of intracoronary and endogenously mobilized bone marrow stem cells in the treatment of ST-segment elevation myocardial infarction: a meta-analysis of randomized control trials. Eur J Heart Fail 2012;14:91-105.

Zohlnhöfer D, Dibra A, Koppara T, de Waha A, Ripa RS, Kastrup J, Valgimigli M, Schömig A, Kastrati A. Stem cell mobilization by granulocyte colony-stimulating factor for myocardial recovery after acute myocardial infarction: a meta-analysis. J Am Coll Cardiol 2008;51:1429-37.

ONLINE TABLES

Table 1S. Included reviews.

First author (acronym)	Journal	Journal impact factor	Year of publication	PubMed ID	Studies included	Patients included
Abdel-Latif	Arch Intern Med	>10	2007	17533201	18	999
Alvarez	Clin Res Cardiol	1-5	2012	23052331	35	2472
Bai	Ageing Res Rev	5-10	2010	20471500	10	850
Brunskill	Eur J Heart Fail	5-10	2009	19654139	21	1091
Cheng	Heart Lung Circ	1-5	2013	23806195	5	210
Clifford	PLoS One	1-5	2012	22655042	33	1765
de Jong	Circ Cardiovasc Interv	5-10	2014	24668227	30	2037
Delewi	Heart	5-10	2012	22875736	24	1624
Delewi	Eur Heart J	>10	2013	24026778	16	1641
Donndorf	J Thorac Cardiovasc Surg	1-5	2011	21376346	6	179
Fan	Cardiovasc Drugs Ther	1-5	2007	18000749	6	320
Fisher	PLoS One	1-5	2013	23840302	9	659
Fisher	Cochrane Database Syst Rev	5-10	2014	24777540	23	1255
Henry	J Am Coll Cardiol	>10	2007	17825712	2	532
Hristow	J Cell Mol Med	1-5	2006	16989732	5	482
Ince	Heart	5-10	2007	17761504	7	325
Jeevanantham	Circulation	>10	2012	22730444	50	2625
Jiang	Expert Opin Biol Ther	1-5	2010	20384520	18	980
Jiang	Stem Cell Res	1-5	2012	22640927	6	365
Kandala	Am J Cardiol	1-5	2013	23623290	10	519
Kang	Clin Ther	1-5	2007	18158081	7	364
Kang	Coron Artery Dis	1-5	2008	18607170	6	517
Kuswardhani	Acta Med Indones	<1	2011	21979282	10	906
Li	Can J Cardiol	1-5	2013	23465346	5	381
Lipinski	J Am Coll Cardiol	>10	2007	17964040	10	698
Martin-Rendon	Eur Heart J	>10	2008	18523058	13	811
Moazzami	Cochrane Database Syst Rev	5-10	2013	23728682	7	354
Nowbar (DAMASCENE)	BMJ	>10	2014	24778175	49	NA
Singh	Clin Cardiol	1-5	2009	19353705	7	516
Sun	Clin Cardiol	1-5	2010	20513068	11	832
Takagi	Int J Cardiol	5-10	2011	21641056	15	877
Tian	Atherosclerosis	1-5	2014	24530783	11	492

Wen	Expert Opin Biol Ther	1-5	2011	21388335	8	307
Wen	Int J Clin Pract	1-5	2011	21762310	7	343
Xu	Can J Cardiol	1-5	2014	24726092	19	886
Zhang	Clin Cardiol	1-5	2009	19685520	7	660
Zhang	Int J Cardiol	5-10	2009	18644638	6	525
Zhang	Clin Cardiol	1-5	2010	20556805	8	725
Zhao	Expert Opin Biol Ther	1-5	2011	21981749	10	422
Zimmet	Eur J Heart Fail	5-10	2011	22065869	29	1830
Zohlnhöfer	J Am Coll Cardiol	>10	2008	18402895	10	445

Table 2S. Additional features of included reviews.

First author (acronym)	Year of publication	Included studies	Setting	Therapy	Country of corresponding author	Number of authors
Abdel-Latif	2007	Non-RCTs and RCTS	IHD/HF	BMSC	USA	9
Alvarez	2012	Non-RCTs and RCTS	IHD/HF	Any type of SC	USA	9
Bai	2010	RTCs	AMI	BMSC	China	3
Brunskill	2009	RTCs	IHD/HF	BMSC	UK	5
Cheng	2013	RTCs	IHD/HF	Any type of SC	China	3
Clifford	2012	RTCs	AMI	BMSC	UK	8
de Jong	2014	RTCs	AMI	BMSC	Netherlands	5
Delewi	2012	RTCs	AMI	BMSC	Netherlands	6
Delewi	2013	RTCs	AMI	BMSC	Netherlands	26
Donndorf	2011	Non-RCTs and RCTS	IHD/HF	BMSC	Germany	7
Fan	2007	RTCs	AMI	G-CSF	China	4
Fisher	2013	RTCs	IHD/HF	BMSC	UK	5
Fisher	2014	RTCs	IHD/HF	BMSC	UK	6
Henry	2007	RTCs	IHD/HF	Other	USA	8
Hristow	2006	RTCs	AMI	BMSC	Germany	4
Ince	2007	RTCs	AMI	G-CSF	Germany	8
Jeevanantham	2012	Non-RCTs and RCTS	IHD/HF	BMSC	USA	6
Jiang	2010	RTCs	IHD/HF	Any type of SC	China	14
Jiang	2012	RTCs	AMI	BMSC	China	3
Kandala	2013	RTCs	IHD/HF	BMSC	USA	6
Kang	2007	RTCs	IHD/HF	G-CSF	China	4
Kang	2008	RTCs	AMI	BMSC	China	4
Kuswardhani	2011	RTCs	AMI	BMSC	China	4
Li	2013	RTCs	IHD/HF	Any type of SC	China	6
Lipinski	2007	RTCs	AMI	BMSC	Italy	11
Martin-Rendon	2008	RTCs	AMI	BMSC	UK	6
Moazzami	2013	RTCs	AMI	G-CSF	USA	3
Nowbar (DAMASCENE)	2014	RTCs	IHD/HF	BMSC	UK	9
Singh	2009	RTCs	AMI	BMSC	USA	7
Sun	2010	Non-RCTs and RCTS	AMI	Any type of SC	China	4
Takagi	2011	RTCs	AMI	Any type of SC	Japan	2
Tian	2014	RTCs	IHD/HF	BMSC	China	5
Wen	2011	RTCs	IHD/HF	BMSC	China	5
Wen	2011	RTCs	IHD/HF	BMSC	China	4

Xu	2014	RTCs	IHD/HF	BMSC	China	5
Zhang	2009	RTCs	AMI	BMSC	China	9
Zhang	2009	RTCs	AMI	BMSC	China	7
Zhang	2010	RTCs	AMI	BMSC	China	9
Zhao	2011	RTCs	IHD/HF	BMSC	China	2
Zimmet	2011	RTCs	AMI	2	Australia	8
Zohlnhöfer	2008	RTCs	AMI	G-CSF	Germany	9

AMI=acute myocardial infarction; BMSC=bone marrow-derived stem cell; G-CSF=granulocyte-colony stimulating factor; HF=heart failure; IHD=ischemic heart disease; Non-RCTs=non-randomized clinical trials; RCTs=randomized clinical trials; SC=stem cell

Table 3S. Findings, effect estimates and citations of included reviews.

First author (acronym)	Year of publication	AMSTAR score	Findings	Effect estimates for LVEF	Effect estimates for death	Yearly WOS citations
Abdel-Latif	2007	10	BMSC are associated with improvements in cardiac function parameters in patients with acute and chronic IHD.	3.66 (1.93 to 5.40)	NA	68,9
Alvarez	2012	3	Most adverse events associated with cell therapy occur during intracoronary infusion and G-CSF administration.	NA	NA	0,0
Bai	2010	9	BMSC are associated with improvements in cardiac function parameters in patients with AMI, especially in the elderly, diabetics, and women.	3.79 (2.4 to 5.7)	NA	1,8
Brunskill	2009	10	BMSC are associated with improvements in cardiac function parameters in patients with AMI, especially when delivered with intramyocardial injection and with lower baseline LVEF.	3.71 (2.15 to 5.26)	NA	5,1
Cheng	2013	9	Stem cells are not associated with changes in prognosis or cardiac function parameters, but may improve symptoms.	0.02 (-0.31 to 0.35)	RR=2.27 (0.60-8.62)	1,4
Clifford	2012	11	BMSC effects on cardiac function parameters seem to be greater in patients with AMI when more than 10^8 cells are injected and with lower baseline LVEF.	3.26 (1.12 to 4.40)	RR=0.70 (0.40-1.21)	6,9
de Jong	2014	10	BMSC are not associated with significant effects on prognosis, or MRI-derived cardiac function parameters.	2.10 (0.68 to 3.52)	OR=0.68 (0.36-1.31) for BMMNC, OR=0.50 (0.09-2.67) for BM progenitor cells, and OR=3.18 (0.13-81.01) for MSC	NA
Delewi	2012	10	BMSC are associated with improvements in prognosis and cardiac fucntion parameters.	2.23 (1.00 to 3.47)	RR=0.60 (0.38-1.08)	3,5
Delewi	2013	#RIF!	BMSC are associated with improvements in cardiac function parameters, especially in younger patients and those with lower baseline LVEF.	2.55 (1.83 to 3.26)	NA	6,8
Donndorf	2011	10	BMSC are associated with improvements in cardiac function parameters in patients undergoing CABG.	5.40 (1.36 to 9.44)	NA	6,2
Fan	2007	10	G-CSF is not associated with changes in cardiac function parameters in patients with AMI.	2.27 (-3.41 to 7.94)	NA	2,0
Fisher	2013	9	BMSC are associated with improvements in prognosis, cardiac function parameters, and symptoms.	3.47 (1.88 to 5.06)	RR=0.33 (0.17-0.65)	4,3

Fisher	2014	11	According to studies with average to low internal validity, BMSC appear to improve prognosis, cardiac function, and symptoms of chronic heart disease, but effects vary substantially depending on route, baseline LVEF, cell type, and setting.	4.22 (3.47 to 4.97)	RR=0.28 (0.14-0.53)	NA
Henry	2007	5	Ad5FGF-4 has no overall impact on signs and symptoms in patients with refractory angina, but gender differences may be present.	NA	NA	10,2
Hristow	2006	4	BMSC are associated with improvements in cardiac function parameters in patients with AMI.	4.21 (0.21 to 8.22)	NA	5,5
Ince	2007	5	G-CSF is not associated with an increase risk of stent restenosis or thrombosis in patients with AMI.	NA	NA	2,8
Jeevanantham	2012	9	BMSC are associated with improvements in prognosis and cardiac fucntion parameters.	3.96 (2.90 to 5.02)	OR=0.39 (0.27 to 0.55)	41,7
Jiang	2010	9	Stem cells are associated with improvements in cardiac function parameters in patients with AMI.	2.93 (2.05 to 3.81)		3,5
Jiang	2012	10	Intracoronary BMSC are associated with improvements in diastolic function parameters and exercise testing features.	NA	NA	0,0
Kandala	2013	9	BMSC is associated with improvements in cardiac function parameters, especially when administered with intramyocardial injection.	4.48 (2.43 to 6.53)	NA	5,1
Kang	2007	8	G-CSF is associated with improvements in cardiac function parameters in patients with AMI.	3.46 (0.60 to 6.32)	NA	2,6
Kang	2008	9	BMSC are associated with improvements in cardiac function parameters in patients with AMI.	2.88 (1.69 to 4.08)	NA	6,2
Kuswardhani	2011	9	BMSC is associated with improvements in prognosis and cardiac function parameters in patietns with AMI.	3.46 (0.60 to 6.32)	NA	NA
Li	2013	10	Stem cells are associated with improvements in prognosis and symptoms.	NA	OR=0.33 (0.08-1.39)	2,4
Lipinski	2007	10	BMSC are associated with improvements in prognosis and cardiac function parameters in patients with AMI, with meta-regression suggesting an association between inject cell volume and LVEF change.	2.97 (1.88 to 4.06)	OR=0.52 (0.16-.63)	43,4

Martin-Rendon	2008	10	BMSC are associated with improvements in cardiac function parameters in patients with AMI.	2.99 (1.26 to 4.72)	RR=0.62 (0.22-1.76)	38,3
Moazzami	2013	11	G-CSF is not associated with changes in prognosis, cardiac function parameters, or symptoms.	3.41 (-0.61 to 7.44)	RR=0.64 (0.15 to 2.80)	0,0
Nowbar (DAMASCENE)	2014	10	The effect of BMSC on LVEF is associated with number of discrepancies in study reports: studies without discrepancies suggest no clinically relevant effect on LVEF.	2.91 (1.47 to 4.35)	NA	NA
Singh	2009	9	BMSC are associated with improvements in cardiac function parameters in patients with AMI.	6.11 (2.67 to 9.54)	NA	4,8
Sun	2010	9	Stem cells are associated with improvements in cardiac function parameters in patients with AMI.	NA	NA	3,5
Takagi	2011	6	Stem cells are not associated with improvements in cardiac function parameters in patients with AMI.	2.87 (1.95 to 3.78)	NA	0,3
Tian	2014	9	BMSC are associated with improvements in cardiac function parameters, especially in subjects with revascularizable regions.	4.91 (2.84-6.99)	NA	NA
Wen	2011	9	BMSC are associated with improvements in cardiac function parameters.	3.83 (2.10 to 5.56)	NA	4,1
Wen	2011	8	BMSC are associated with improvements in cardiac function parameters.	3.72 (1.98 to 5.46)	NA	1,5
Xu	2014	9	BMSC are associated with improvements in prognosis and cardiac fucntion parameters in patients with chronic IHD and HF.	3.54 (1.92 to 5.17)	RR=0.49 (0.29-0.84)	NA
Zhang	2009	9	BMSC transfer 4-7 days post-AMI is associated with improvements in prognosis and cardiac function parameters.	4.63 (1.00 to 8.26)	RR=0.33 (0.09-1.22)	5,1
Zhang	2009	9	BMSC are associated with improvements in cardiac function parameters in patients with AMI.	4.77 (1.42 to 8.12)	NA	3,6
Zhang	2010	9	BMSC are associated with improvements in prognosis and cardiac fucntion parameters, especially in younger patients and those receiving BMSC 6-7 days post-AMI.	4.37 (2.66 to 6.08)	RR=0.33 (0.13-0.89)	2,7
Zhao	2011	9	BMSC are associated with improvements in cardiac function parameters which may depend on primary intervention, route, cell type, and baseline LVEF.	4.59 (2.22 to 6.95)	NA	0,6

Zimmet	2011	9	BMSC and G-CSF are associated with improvements in prognosis and cardiac function parameters.	2.70 (1.48 to 3.92) for BMSC, and 2.81 (-1.49 to 7.11) for G-CSF	OR=1.16 (0.40-3.57) for BMSC, and OR=1.69 (0.19-20.5) for G-CSF	7,6
Zohlnhöfer	2008	7	G-CSF is not associated with changes in cardiac function parameters in patients with AMI.	1.32 (-1.52 to 4.16)	NA	11,4

ONLINE FIGURES

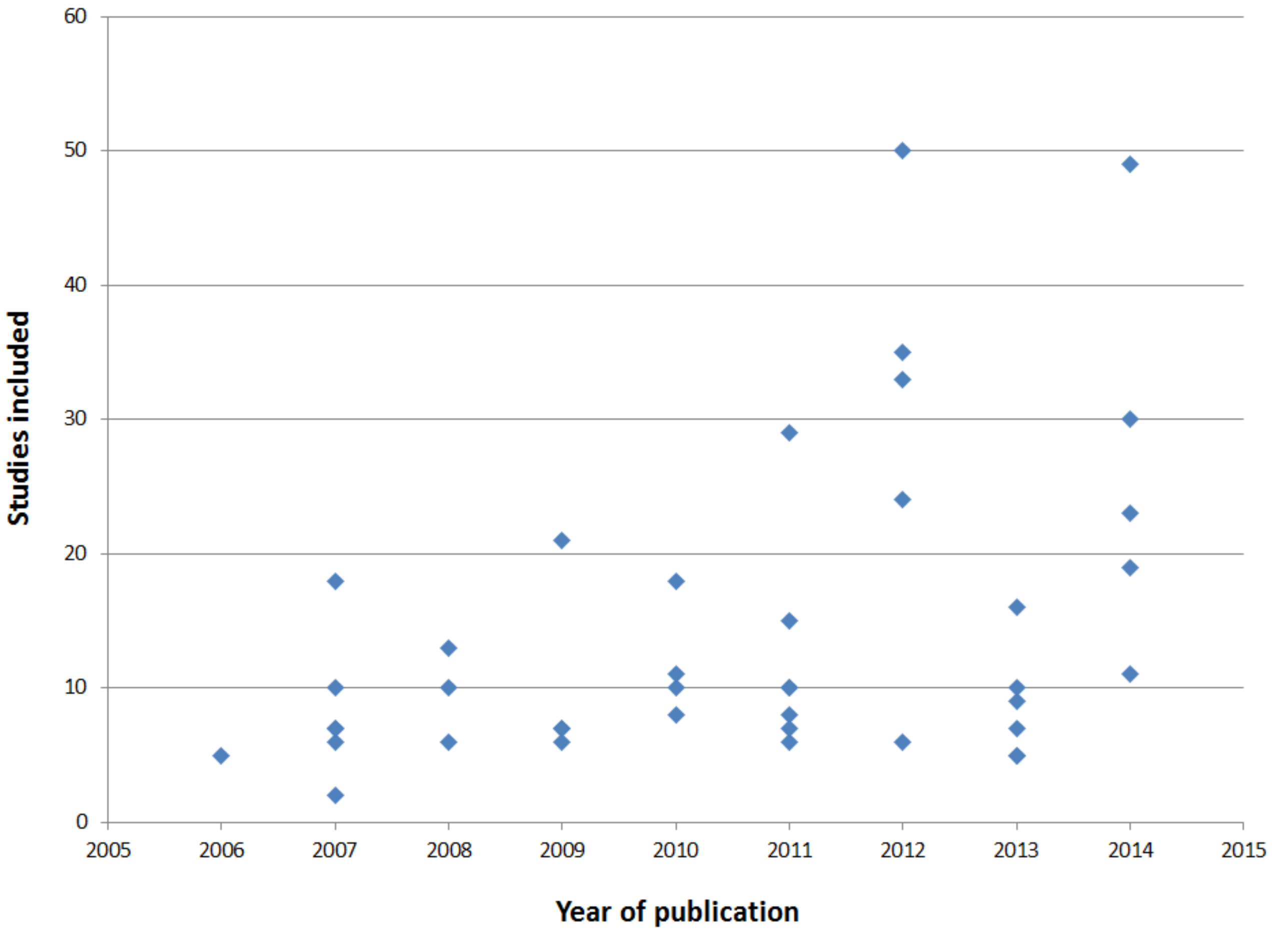

Figure 1S. Trend in the number of included studies over time. Each square represents a single systematic review.
